# Colchicin: altes Medikament mit neuem Nutzen: Einsatz in der Rheumatologie und darüber hinaus

**DOI:** 10.1007/s00393-021-01017-z

**Published:** 2021-06-07

**Authors:** Z. Boyadzhieva, N. Ruffer, M. Krusche

**Affiliations:** 1 Medizinische Klinik mit Schwerpunkt Rheumatologie und Klinische Immunologie, Charité Universitätsmedizin, Charitéplatz 1, 10117 Berlin, Deutschland; 2 Abteilung für Rheumatologie und Immunologie, Klinikum Bad Bramstedt, Bad Bramstedt, Deutschland

**Keywords:** Herbstzeitlose, Autoinflammation, Neutrophile Granulozyten, Kardiovaskuläre Erkrankung, COVID-19, Autumn crocus, Autoinflammation, Neutrophil granulocytes, Cardiovascular disease, COVID-19

## Abstract

Colchicin, das Gift der Herbstzeitlosen, hat verschiedene antiinflammatorische Effekte. Aus diesem Grund kommt es zur Behandlung von rheumatologischen Erkrankungen aus dem autoinflammatorischen Formenkreis, wie z. B. der Arthritis urica oder dem familiären Mittelmeerfieber (FMF), zum Einsatz. Darüber hinaus gibt es erste Daten, die einen positiven Nutzen von Colchicin bei kardiovaskulären Erkrankungen nahelegen. Des Weiteren werden aktuell verschiedene antiinflammatorische Therapieansätze in der COVID-19-Behandlung in Studien erprobt. Hier gibt es ebenfalls erste Publikationen, die einen potenziellen Nutzen von Colchicin in bestimmten Krankheitsphasen der Virusinfektion nahe legen. Dieser Beitrag will einen Überblick über die Wirkweise, den Nutzen und Nebenwirkungen sowie die verschiedenen Einsatzmöglichkeiten von Colchicin in der Rheumatologie geben. Weiterhin soll ein kurzer Ausblick in neue Einsatzgebiete dieses Medikamentes gegeben werden.

## Abstract (en)

Colchicine, the toxin of the autumn crocus, has various anti-inflammatory effects. For this reason, it is being used for the treatment of several autoinflammatory diseases, such as gout or familial Mediterranean fever (FMF). In addition, some interesting studies have been published which suggest the benefits of colchicine in cardiovascular diseases. Furthermore, various anti-inflammatory therapeutic approaches are currently being tested in clinical trials for the treatment of COVID-19. First publications suggest a potential benefit of colchicine in certain disease phases of the virus infection. This article provides an overview of the mechanisms of action, benefits and side effects as well as the various possible uses of colchicine in rheumatology. Furthermore, a brief preview of potential new areas for use of the drug, which are also of interest to rheumatologists, are presented.

Colchicin ist ein trizyklisches, lipidlösliches Alkaloid mit antiinflammatorischer Wirkung.

Der Name leitet sich von dem georgischen Königreich Kolchis ab, welches in der Argonauten-Saga beschrieben wird. Der griechischen Mythologie nach soll sich in Kolchis ein berühmter Garten mit Heil- und Giftpflanzen befunden haben.

Die erstmalige Erwähnung des medizinischen Einsatzes von Colchicin reicht bis in das alte Ägypten zurück. Im Papyrus Ebers wurde bereits über den Einsatz von Colchicin zur Behandlung von Gichtanfällen berichtet.

Die Entdeckung des Alkaloids gelang Pellentier und Caventou 1819. Bis zur erstmaligen biochemischen Totalsynthese von Colchicin durch Albert Eschenmoser 1959 wurde Colchicin aus der Herbstzeitlosen (*Colchicum autumnale*) (Abb. 1) und der Ruhmeskrone (*Gloriosa superba*) gewonnen.

**Figure 1:**
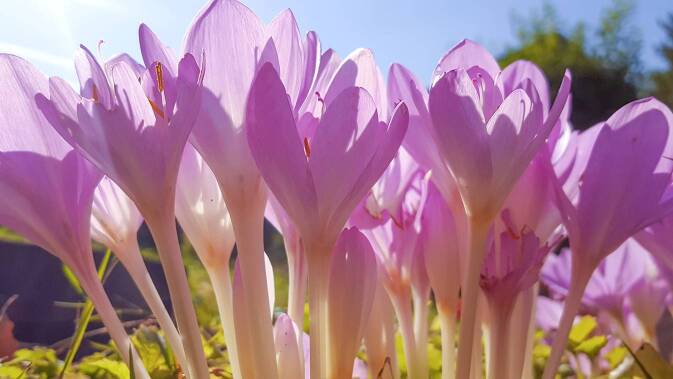

## Wirkmechanismus

Colchicin wirkt über eine Hemmung der Polymerisation von Mikrotubuli antimitotisch, weshalb das Medikament umgangssprachlich auch als „Spindelgift“ bezeichnet wird. Darüber hinaus hat es eine antiinflammatorische Wirkung, die über zahlreiche direkte und indirekte Effekte auf das angeborene Immunsystem vermittelt wird.

Die Polymerisationshemmung beeinflusst maßgeblich neutrophile Granulozyten. Hierbei wird dosisabhängig über die verminderte Expression von E‑Selektin auf Endothelzellen die Adhäsion und Migration von Neutrophilen in entzündetes Gewebe gehemmt [1]. Außerdem fördert Colchicin den Abbau von L‑Selektin auf der Oberfläche von Neutrophilen. L‑Selektin wird für die Extravasation zirkulierender Leukozyten benötigt. Durch seinen Abbau wird die weitere Neutrophilenrekrutierung gehemmt. Die Motilität und Verformbarkeit von Neutrophilen, essenziel für deren erfolgreiche Migration und Extravasation, werden ebenfalls durch Colchicin gestört [2, 3].

Weiterhin ist Colchicin in der Lage, die Formation und Aktivierung des NLRP3 Inflammasoms als zentrale Schaltstelle für verschiedene autoinflammatorische Prozesse (wie z. B. der Gicht oder dem familiären Mittelmeerfieber [FMF]) zu blockieren. Die exakten Wirkmechanismen hierfür sind immer noch Gegenstand der aktuellen Forschung. Es konnte aber u. a. bereits nachgewiesen werden, dass Colchicin die Entstehung der P2X7- und P2X2-Poren inhibiert, die wiederum ATP-abhängig das NLRP3-Inflammasom aktivieren. Darüber hinaus hemmt Colchicin direkt die Freisetzung von TNF‑α, Stickstoffmonooxid und reaktiven Sauerstoffspezies (ROS) [4].

## Unerwünschte Arzneimittelwirkungen (UAW)

Die häufigsten Nebenwirkungen des Medikaments erklären sich durch seinen Wirkmechanismus – die Hemmung der Polymerisation der Mikrotubuli. Da diese essenziell für den Aufbau der Mitosespindel und somit für eine regelrechte Zellteilung ist, werden zuerst Gewebe mit einer hohen Proliferationsrate beeinflusst – wie der Gastrointestinaltrakt und das Knochenmark.

Eine aktuelle Metaanalyse von Stewart et al. konnte zeigen, dass Diarrhöen als häufigste UAW bei ca. 18 % der Patienten auftreten [5]. Fast gleich häufig wird über Übelkeit, Erbrechen und abdominelle Schmerzen berichtet. Die gastrointestinalen Effekte treten dosisabhängig auf und sind nach Dosisreduktion reversibel, wie die randomisierte placebokontrollierte AGREE-Studie zeigen konnte [6]. Diese untersuchte, ob Colchicin effektiv zur Schmerzreduktion 24 h nach Beginn eines akuten Gichtanfalls war. Im Colchicin-Arm bekamen die Patienten entweder insgesamt 1,8 mg (Einmalgabe 1,2 mg und 0,6 mg nach 1 h) oder 4,8 mg (1,2 mg und 0,6 mg stündlich über 6 h) Colchicin. Während bei niedriger Dosierung 23 % der Patienten Diarrhöen entwickelten, zeigten dies fast 77 % der Patienten mit hoch dosierter Colchicin-Therapie. Schwere Durchfälle und Erbrechen traten sogar nur in der zweiten Gruppe auf. Interessanterweise zeigte sich in der Metaanalyse von Stewart et al. kein erhöhtes Infektionsrisiko. Hepatische UAW (erhöhte Transaminasen) wurden selten berichtet (1,9 %).

Eine Knochenmarkdepression kann sich als Thrombozytopenie, Agranulozytose oder aplastische Anämie bemerkbar machen. Bei Beachten der empfohlenen Colchicin-Tagesdosis ist diese sehr selten – in der Metaanalyse von Stewart et al. wird auch hierfür kein erhöhtes Risiko beschrieben – insgesamt 0,4 % der Patienten hatten Blutbildveränderungen [5].

Ebenfalls selten (insgesamt 0,8 %) sind UAW der Skelettmuskulatur (Krämpfe, Schwäche, Myalgien, Erhöhung der Kreatinkinase[CK]-Werte) beschrieben worden [5]. Mit dem gehäuften Auftreten von muskulären Symptomen ist unter Colchicin bei normaler Nierenfunktion (renale Elimination von Colchicin) nicht zu rechnen. Einzelne Fallberichte diskutieren eine durch Colchicin verursachte Neuromyopathie mit proximaler Schwäche, axonaler Polyneuropathie und CK-Anstieg, die sich auf dem Hintergrund einer Niereninsuffizienz entwickelte [7, 8]. Es bestehen auch Hinweise auf einen Zusammenhang mit einer gleichzeitigen Statintherapie, da manche Statine auch über das CYP3A4-Enzymsystem metabolisiert werden und es somit zur Akkumulation von Colchicin kommen kann [9, 10]. Die Metaanalyse von Stewart et al. konnte jedoch kein erhöhtes Risiko für eine Neuromyopathie unter Colchicin-Therapie identifizieren.

## Überdosierung

Insgesamt weist Colchicin eine enge therapeutische Breite auf. Allerdings ist es schwer, einen eindeutigen Cut-off-Wert zwischen nichttoxischer, toxischer und letaler Dosis festzulegen. Es gibt Fallberichte, die nahelegen, dass eine akute Einnahme von über 0,5 mg/kgKG mit erhöhter Mortalität assoziiert ist, letale Verläufe sind u. a. auch bei relativ niedrigen Dosierungen zwischen 7 und 26 mg beschrieben worden [11].

Der Verlauf einer sog. Colchicin-Vergiftung kann grob in 3 Phasen eingeteilt werden: In der initialen gastrointestinalen Phase kommt es innerhalb 10–24 h nach Einnahme zum Auftreten von abdominellen Krämpfen, Durchfällen, Übelkeit, Erbrechen bis hin zu hämorrhagischen Enteritiden. Die zweite Phase (1 bis 7 Tage nach Einnahme) ist gekennzeichnet durch eine Multiorgandysfunktion mit schwerer Knochenmarkdepression, akutem Leber- und/oder Nierenversagen, Herzrhythmusstörungen, Hämolyse, hämorrhagischen Komplikationen und metabolischen Störungen wie metabolischer Azidose und Elektrolytentgleisungen. Ursächlich für einen letalen Ausgang können ein hämodynamischer Kollaps oder schwerwiegende Infektionen sein. Die dritte sog. Erholungsphase setzt nach 7 bis 21 Tagen ein. Hier kann sich laborchemisch eine Rebound-Leukozytose zeigen, die Symptomatik normalisiert sich insgesamt wieder [11].

Das Management der Colchicin-Vergiftung ist rein supportiv. In der Initialphase können eine Magenspülung und die Gabe von Aktivkohle sinnvoll sein. Wichtig ist, dass aufgrund des hohen Verteilungsvolumens von Colchicin eine Hämodialyse nicht effektiv ist [11, 12].

In der Literatur findet man einen einzelnen Fallbericht über den experimentellen Einsatz von Colchicin-spezifischen Fab-Antikörpern bei einer akuten Vergiftung beim Menschen. Eine junge Patientin wurde nach Einnahme von 60 mg Colchicin erfolgreich behandelt [13]. Die Wirksamkeit der Fab-Antikörper zur Steigerung der Clearance von Colchicin wurde neulich im Tiermodell nachgewiesen, klinische Studien sind noch zu erwarten [14, 15].

## Pharmakokinetik

### Metabolismus

Die Halbwertszeit von Colchicin beträgt ca. 20–40 h. Colchicin wird hepatisch durch das CYP3A4-Isoenzym metabolisiert, das zu dem Cytochrom-P450-Enzymsystem gehört. Dieses ist eine zentrale Schaltstelle der Verstoffwechselung verschiedener, körperfremden Stoffe, wobei CYP3A4 am meisten Substrate aufweist. Darüber hinaus ist Colchicin auch Substrat vom P‑Glykoprotein-Efflux-Transporter – ein membranständiges Protein, das wiederum körperfremde Stoffe aus den Zellen abtransportiert. Weiterhin werden 10–20 % des Medikamentes renal eliminiert, sodass eine Dosisanpassung bei schwerer Niereninsuffizienz (glomeruläre Filtrationsrate [GFR] < 30 ml/min) empfohlen wird [16].

### Interaktionen

Da Colchicin über CYP3A und dem P‑Glykoprotein metabolisiert wird, ergibt sich eine Vielzahl an möglichen relevanten Arzneimittelwechselwirkungen (vgl. Tab. 1). Deshalb wird bei gleichzeitiger Therapie mit diesen Medikamenten eine vorsichtige Dosisanpassung bzw. die Wahl eines anderen Präparates angeraten. So wird unter anderem bei der Indikation für eine Statintherapie empfohlen, dass ein Statin gewählt werden sollte, das nicht über das CYP3A4-Isoenzym metabolisiert wird (z. B. Pravastatin oder Rosuvastatin) [9, 10].

**Table 1:** 

Substanzklasse	CYP3A4-Inhibitoren	P‑Glykoprotein-Inhibitoren
Antiarrhythmika	Amiodaron	Amiodaron, Chinidin, Chinin, Dronedaron, Propafenon
Antibiotika	Clarithromycin, Erythromycin	
Antimykotika	Fluconazol, Itraconazol, Ketoconazol, Voriconazol	Itraconazol, Ketoconazol
Kalziumkanalblocker	Diltiazem, Verapamil	Verapamil
Proteaseinhibitoren	Indinavir, Lopinavir	Lopinavir
Immunsuppressiva	–	Cyclosporin A
Statine	–	Atorvastatin, Simvastatin
Malariamedikamente	–	Mefloquin
Sonstige	Grapefruitsaft	–

Besondere Vorsicht ist weiterhin bei der Komedikation mit Clarithromycin geboten: Zapata et al. konnten in dem Meldesystem für UAW der FDA (FAERS) 58 Patientenfälle mit einer Colchicin-Überdosierung bei paralleler Clarithromycin-Gabe identifizieren. Darüber hinaus berichteten die Autoren über 20 in der Literatur publizierte Fälle einer solchen Arzneimittelinteraktion, die meist zur Häufung der UAW und in 4 der 20 Fälle zum Tode führte [17].

## Kontraindikationen

### Chronische Niereninsuffizienz

Bei Patienten mit chronischer Niereninsuffizienz ist Vorsicht geboten. Wegen der verringerten Clearance kann es zur Akkumulation von Colchicin kommen und somit zur Häufung von UAW. Insofern sollte bei höhergradig eingeschränkter Nierenfunktion eine genaue Risiko-Nutzen-Abwägung getroffen werden. Angemessen ist eine Laborbestimmung von sog. Sicherheitsparametern (z. B. CK, Kreatinin, Differenzialblutbild), um UAW zu erkennen [18].

Die aktuelle EULAR-Leitlinie für die Gichttherapie empfiehlt, Colchicin in der Akuttherapie von Patienten mit höhergradiger Niereninsuffizienz (GFR < 30 ml/min) zu meiden, und sieht eine Dosisreduktion für die Anfallsprophylaxe vor [19]. Für FMF-Patienten, die erhöhte CK-Werte aufweisen, wird ebenfalls eine Dosisreduktion empfohlen [20].

### Leberfunktionsstörung

In seltenen Fällen wird über eine Erhöhung der Transaminasen als Nebenwirkung einer Colchicin-Therapie berichtet. Aufgrund der hepatischen Metabolisierung kann eine Leberfunktionsstörung zur Erhöhung der Colchicin-Serumspiegel führen. Verschiedene Tiermodelle und Studien bei Patienten mit einer Leberfunktionsstörung liefern Hinweise dafür, dass Colchicin bei Leberinsuffizienz langsamer eliminiert wird [21, 22]. Eine regelmäßige Kontrolle von Glutamat-Pyruvat-Transaminase (GPT) und Gamma-Glutamyltransferase (γ-GT) wird daher empfohlen.

### Schwangerschaft und Kinderwunsch

Da Colchicin als Mitosehemmstoff gilt und dazu auch noch plazentagängig ist, war lange Zeit unklar, ob das Medikament embryotoxisch ist und ob ein Zusammenhang mit Fehlgeburten besteht. Eine Metaanalyse von Indraratna et al. konnte jedoch keine Erhöhung der Fehlbildungsrate oder eine erhöhte Fehlgeburtenrate nachweisen [23]. Eine 2021 publizierte Metaanalyse von Carnovale et al. untersuchte den Einfluss von Colchicin auf Schwangerschaften und genauer auf die Rate von Schwangerschaftsabbrüchen, Fehlgeburten, kongenitalen Malformationen, Präeklampsie und Frühgeburten. Hier konnten ebenfalls keine signifikanten Unterschiede zwischen Schwangerschaften von mehr als 1000 mit Colchicin behandelten FMF-Patientinnen und gesunden Frauen gezeigt werden [24].

Ein Anstieg der Häufigkeit chromosomaler Anomalien bei Kindern konnte bisher nicht beobachtet werden – weder bei väterlicher noch bei mütterlicher Colchicin-Einnahme. Die Therapie mit Colchicin kann daher auch in der Schwangerschaft fortgeführt werden. Prospektive Studien bei stillenden Frauen fanden bisher keine Auffälligkeiten in der Entwicklung der gestillten Kinder [25]. Männern wird bei bestehendem Kinderwunsch ebenfalls kein Absetzen von Colchicin empfohlen. Kleine Studien und Fallberichte zeigten Auffälligkeiten der Spermienqualität bei Patienten unter Colchicin-Therapie, ein eindeutiger Zusammenhang mit Colchicin konnte bisher nicht nachgewiesen werden.

Der erste Fallbericht über Azoospermie bei einem Patienten unter Colchicin-Therapie wurde 1972 publiziert. Merlin et al. berichteten über einen Gichtpatienten mit reversibler Azoospermie unter Colchicin-Dauertherapie [26]. Darüber hinaus zeigten Spermiogramme bei FMF-Patienten nur in 40 % einen Normalbefund [27]. Eine Studie über die Spermienqualität von 62 Patienten mit Behçet-Syndrom zeigte eine Oligo- oder Aspermie in 40 % der Patienten [28]. Allerdings konnte in keiner Studie der Zusammenhang zur Therapie mit Colchicin bewiesen werden. Manche Autoren spekulieren, dass die Grunderkrankung selbst eine Rolle spielen könnte [29]. Eine Studie von Ben-Chetrir et al. verfolgte darüber hinaus Schwangerschaften von Frauen, deren Partner FMF-Patienten unter Colchicin-Therapie waren, und verglich diese mit Schwangerschaften von gesunden Frauen mit gesunden Partnern. Es zeigte sich kein erhöhtes Risiko für einen Schwangerschaftsabbruch oder für kongenitale Malformationen [30].

Laut den EULAR-Empfehlungen von 2016 ist Colchicin mit einer Schwangerschaft kompatibel. Es sollte daher zum Remissionserhalt und zur Therapie der zugrunde liegenden rheumatologischen Erkrankung in der Schwangerschaft fortgeführt werden. Eine Gabe während der Stillzeit sollte in Erwägung gezogen werden, solange vonseiten des gestillten Kindes keine Kontraindikationen bestehen [31].

## Indikationen, Anwendungsgebiete und Dosierung

### Gicht

Gemäß den aktuellen EULAR-Leitlinien für die Gichttherapie ist Colchicin den nichtsteroidalen Antirheumatika (NSAR) und oralen Kortikosteroiden als Erstlinientherapie gleichgesetzt [19]. Die optimale Dosierung von Colchicin, insbesondere in der Initialphase eines Gichtanfalls, ist aktuell Gegenstand der Forschung: Die AGREE-Studie konnte zeigen, dass eine niedrig dosierte Colchicin-Gabe (Einmalgabe 1,2 mg und 0,6 mg nach 1 h) innerhalb von 12 h nach Anfallsbeginn Schmerzen ebenso effektiv linderte wie eine hoch dosierte Therapie (1,2 mg und 0,6 mg stündlich über 6 h) [6].

In Deutschland ist Colchicin nur in Tablettenform mit einer Dosis von 0,5 mg verfügbar. Die deutsche Leitlinie empfiehlt für den akuten Gichtanfall eine Therapie mit 1 bis 3 Gaben à 0,5 mg pro Tag [32]. In der Leitlinie der EULAR wird hingegen eine Initialdosis von 1 mg, gefolgt von 0,5 mg empfohlen [19]. Eine Gesamtdosis von 6 mg bis zum Rückgang der Symptomatik sollte nicht überschritten werden [12]. Die Einnahme im Anfall sollte bis Abklingen der Symptome weitergeführt werden (in der Regel < 14 Tage). Als Anfallsprophylaxe ist Colchicin 0,5–1 mg/Tag über 3 bis 6 Monate einzunehmen (vgl. Tab. 2).

**Table 2:** 

Indikation	Dosierung	Dauer	Tapering
Gicht	Im Anfall: 0,5 mg 1‑ bis 3‑mal/Tag Anfallsprophylaxe: 0,5 mg/Tag	Im Einzelfall kann die Verordnung über 6 Monate hinaus erfolgen, wenn rezidivierende Gichtanfälle trotz Prophylaxe auftreten [32]	Keine Angaben (k. A.)
FMF	Kinder > 10 J und Erwachsene: 1,5 mg/Tag Dosissteigerung in Schritten von 0,5 mg Colchicin/Tag bis zur Maximaldosis von 2,0 mg Colchicin/Tag im Kindesalter und 3,0 mg Colchicin/Tag im Erwachsenenalter	Lebenslang	Dosisreduktion um 0,5 mg alle 6 Monate möglich bei Fehlen von Schüben > 5 J und normwertigen Entzündungsparametern, solange regelmäßige Laborkontrollen erfolgen [20]
Behçet-Syndrom	2‑mal 0,5 bis 2‑mal 1 mg/Tag	Minimum 3 bis 6 Monate [42]	Je nach klinischer und laborchemischer Krankheitsaktivität ggf. Dosisreduktion um 0,5 mg alle 3 bis 6 Monate
Idiopathisch rekurrierende Perikarditis	2‑mal 0,5 mg/Tag oder 1‑mal 0,5 mg/Tag für Patienten < 70 kg oder mit Intoleranz für höhere Dosen	Mindestens 6 Monate	Nicht nötig, alternativ 0,5 mg jeden zweiten Tag, oder 0,5 mg 1‑malig (Körpergewicht > 70 kg) in den letzten Wochen
PFAPA	0,5–1,25 mg/Tag	Unklar, da Erkrankung meist selbstlimitierend	k. A.

### Familiäres Mittelmeerfieber (FMF)

Colchicin ist ein Grundpfeiler in der Therapie des FMF. Es reduziert nachweislich die Schubfrequenz und senkt das Amyloidoserisiko [33, 34]. Für Erwachsene wird eine Tagesdosis von 1,5 mg/Tag angestrebt [35]. Eine langsame Dosissteigerung kann je nach klinischem Beschwerdebild, Entzündungsparametern und ggf. beim Vorliegen einer Amyloidose (cave: Nierenfunktion) erfolgen. Bei Erwachsenen kann Colchicin bei guter Verträglichkeit (sowie normaler Nierenfunktion) im Falle einer persistierenden klinischen oder serologischen Entzündungsaktivität bis zu einer maximalen Tagesdosis von 3 mg aufdosiert werden. Die kontinuierliche tägliche Einnahme von Colchicin stabilisiert die Proteinurie bei Patienten mit einer manifesten Amyloidnephropathie und kann den Progress einer chronischen Niereninsuffizienz verlangsamen [33, 36].

### Colchicin-Resistenz

Bei unzureichender Krankheitskontrolle des FMF unter Colchicin-Therapie wird der Begriff Colchicin-Resistenz verwendet [37]. Die Begrifflichkeit ist in der Literatur jedoch nicht einheitlich definiert. Laut EULAR-Definition liegt eine Colchicin-Resistenz vor, wenn FMF-Patienten trotz guter Compliance und Therapie mit der maximal tolerierten Dosis über mehr als 6 Monate mindestens 1 Attacke pro Monat haben [20]. Nach Ansicht der Autoren ist die Definition von Erden et al. für eine differenzierte Beurteilung des Therapieerfolges jedoch sinnvoller, da diese neben der Klinik auch laborchemischer Marker mit einbezieht: Eine Colchicin-Resistenz wird hier definiert als mehr als 1 FMF-Schub in 3 Monaten oder Erhöhung mindestens 2 von 3 Entzündungswerten (Blutsenkungsgeschwindigkeit, C‑reaktives-Protein [CRP] oder Serumamyloid A) zwischen den Schüben oder Vorhandensein einer Amyloidose trotz adäquater Einnahme der maximal tolerierten Colchicin-Dosis [37]. Eine Colchicin-Resistenz wird in der Literatur in 5–15 % der Fälle angegeben [38, 39].

### Behçet-Syndrom

Auch für das Behçet-Syndrom ist die antiinflammatorische Therapie mit Colchicin als Basismedikament etabliert. Insbesondere für die Therapie von rekurrierenden oralen und genitalen Ulzerationen ist eine gute Wirksamkeit belegt. Besonders positive Effekte konnten für die Behandlung von dominanten genitalen Ulzera oder für das Erythema nodosum nachgewiesen werden [40]. Darüber hinaus wird zur Arthritistherapie Colchicin als Erstlinientherapie in den EULAR-Leitlinie empfohlen [41]. Die vor Kurzem publizierten französischen Empfehlungen für die Diagnostik und Therapie des Behçet-Syndroms unterstreichen die Rolle von Colchicin zur Erstlinientherapie bei mukokutanen Läsionen und bei Gelenkbeteiligung in einer Dosierung von 1–2 mg/Tag [42].

### Akute Perikarditis und idiopathisch rekurrierende Perikarditis (IRP)

Niedrig dosiertes Colchicin gehört neben ASS oder Ibuprofen zur Erstlinientherapie bei akuter Perikarditis [43]. Die Therapie erfolgt abhängig vom Körpergewicht mit 1‑mal 0,5 mg/Tag oder 2‑mal 0,5 mg/Tag und sollte über 3 Monate durchgeführt werden.

Eine IRP wird definiert als akute Perikarditis, die nach einer dokumentierten früheren Episode nach einem symptomfreien Intervall von mindestens 4 bis 8 Wochen auftritt. In klinischen Studien konnte gezeigt werden, dass Colchicin nachweislich die Behandlungsdauer einer IRP verkürzt, die Remissionsdauer verlängert und das Rezidivrisiko um ca. 50 % senkt [44–46]. Die European Society of Cardiology(ESC)-Leitlinie empfiehlt als Erstlinientherapie der IRP einer Dosierung von 0,5 mg 2‑mal täglich für mindestens 6 Monate [43].

### PFAPA-Syndrom

Das PFAPA-Syndrom ist eine seltene autoinflammatorische Erkrankung, die klinisch durch periodisches Fieber, aphthöse Stomatitis, Pharyngitis und zervikale Adenitis gekennzeichnet ist. Neben NSARs kommt auch Colchicin als antiinflammatorische Therapie zum Einsatz. In einer randomisierten kontrollierten Studie mit 18 pädiatrischen Patienten zeigte Colchicin eine effektive Reduktion der Schubfrequenz [47]. Bei gleichzeitigem Nachweis einer MEFV-Mutation scheint sich dieser Effekt zu verstärken [48, 49].

Ein Konsens bezüglich Dosierung und Therapiedauer besteht nicht, da die Erkrankung in der Regel selbstlimitierend verläuft. Vorgeschlagen wird eine Schubprophylaxe mit 0,5–1,25 mg Colchicin pro Tag [50].

Einen Überblick über die einzelnen Erkrankungen sowie die Dosierung von Colchicin gibt Tab. 2.

## Kardiovaskuläre Erkrankungen

Die Atherosklerose ist eine Erkrankung entzündlicher Genese. Ursächlich für die Inflammation in arteriosklerotischen Plaques sind Lipidablagerungen und die Abwehrreaktionen gegen in den Plaques vorhandene veränderte Proteine: Es bestehen Hinweise dafür, dass Cholesterinkristalle wichtig für die Entwicklung, Progression und Instabilität von atherosklerotischen Plaques sind. Sie können direkt zur Plaqueruptur führen [51] und die IL-1β-abhängige Inflammationskaskade durch Aktivierung des NLRP3-Inflammasoms triggern [52]. Weiterhin konnte in rupturierten Plaques eine höhere Konzentration von neutrophilen Granulozyten nachgewiesen werden, die vermutlich das Risiko für eine Plaqueruptur erhöhen [53].

In diesem Zusammenhang erscheint die Hemmung von IL-1β interessant: Die CANTOS-Studie (randomisierte kontrollierte Studie [RCT]) untersuchte den Effekt einer IL-1β-Blockade (Canakinumab) bei Patienten mit stattgehabtem Myokardinfarkt und inflammatorischer Aktivität. Für die Canakinumab-Gruppe konnte eine signifikante Senkung der Rezidivrate (Myokardinfarkt, Schlaganfall oder kardiovaskulärer Tod) im Vergleich zur Placebogruppe gezeigt werden. Allerdings traten vermehrt Infektionen als Nebenwirkung von Canakinumab auf [54].

Anknüpfend an diese Daten, wird aktuell untersucht, ob Colchicin, das ebenfalls die Formation des NLRP3-Inflammasoms sowie die Neutrophilenfunktion beeinflusst, zur Therapie und Prävention der Atherosklerose sinnvoll sein könnte.

In ersten Studien wurden positive Effekte von Colchicin bei koronarer Herzkrankheit (KHK) nachgewiesen. Die LoDoCo-Studie (multizentrische RCT) konnte bei 532 Patienten mit stabiler KHK zeigen, dass Colchicin in einer Dosierung von 0,5 mg/Tag zusätzlich zur Sekundärprävention (mit Statinen, ASS und/oder Clopidogrel) das Risiko für kardiovaskuläre Ereignisse senkt. Ein akutes Koronarsyndrom (ACS), nichtkardioembolische zerebrale Ischämien oder ein Herzstillstand traten bei nur 5,3 % in der Colchicin-Gruppe und bei 16 % in der Kontrollgruppe auf [55].

Die LoDoCo2-Studie (multizentrische RCT) konnte ebenfalls eine Risikosenkung durch Colchicin bei chronischer KHK zeigen. Die tägliche Colchicin-Einnahme (0,5 mg/Tag) führte zur Senkung des relativen Risikos für kardiovaskulären Tod, Myokardinfarkt, ischämischen Schlaganfall oder ischämiebedingte Revaskularisierung um 31 % nach median 28 Monaten. Die Häufigkeit einer Hospitalisierung aufgrund von Infektionen, Pneumonie oder Durchfall war in beiden Gruppen häufig [56].

Die COLCOT-Studie (multizentrische RCT) untersuchte den Effekt von Colchicin bei Patienten mit kürzlich stattgehabtem Myokardinfarkt (30 Tage nach akutem Myokardinfarkt). Hierbei senkte die tägliche Gabe von niedrig dosiertem Colchicin (0,5 mg) die Häufigkeit kardiovaskulärer Ereignisse im Vergleich zur Placebogruppe (5,5 % vs. 7,1 %). In der Colchicin-Gruppe traten jedoch signifikant häufiger Pneumonien auf (0,9 % vs. 0,4 %) [57].

Eine prospektive randomisierte Studie von Deftereos et al. lieferte außerdem erste Hinweise dafür, dass Colchicin die Größe eines Myokardinfarkts reduzieren könnte; 151 Patienten mit einem ST-Strecken-Hebungsinfarkt wurden zu Colchicin oder Placebo randomisiert. Vor perkutaner koronarer Intervention (PCI) bekamen 77 Patienten 2 mg Colchicin. Über 5 Tage wurde die Einnahme mit 1 mg/Tag fortgeführt. Bei diesen Patienten zeigten sich laborchemisch eine niedrigere Konzentration von CK sowie niedrigere CRP-Spiegel. Darüber hinaus wurde bei einem Teil der rekrutierten Patienten eine kardiale MRT mit Kontrastmittel durchgeführt. Hier zeigte sich auch eine statistisch signifikant kleinere Infarktgröße in der Interventionsgruppe [58].

Darüber hinaus untersuchen aktuell weitere Studien den möglichen Nutzen von Colchicin zur Therapie weiterer arteriosklerotischer Erkrankungen (wie z. B. Schlaganfall) [59].

Insgesamt erscheint die zurzeit verfügbare Datenlage über Colchicin zur Therapie der KHK vielversprechend, sodass spekuliert werden kann, dass Colchicin zukünftig in den Therapiealgorithmus der KHK mit aufgenommen werden könnte.

## COVID-19

Die SARS-CoV-2-Infektion führt zu einer Aktivierung multipler immunologischer Signalkaskaden [60]. Entscheidend für den Verlauf einer Erkrankung scheint unter anderem das Ausmaß der Aktivierung des NLRP3-Inflammasoms zu sein. Bei einer Hyperaktivierung kommt es zur massiven Freisetzung von „damage-associated molecular patterns“ (DAMPs), Pyroptose, Rekrutierung von Neutrophilen und zur exzessiven Zytokinfreisetzung [61]. Vor diesem Hintergrund werden unterschiedliche antiinflammatorische Therapieansätze für die Behandlung von COVID-19-Patienten untersucht, darunter auch Colchicin.

Interessant erscheinen die vorläufigen Ergebnisse der multizentrischen COLCORONA-Studie, die momentan jedoch nur als Preprint verfügbar sind (Stand 06.04.2021). Die Vorabdaten zeigen, dass Colchicin das Mortalitätsrisiko und das Risiko einer Hospitalisierung im Vergleich zu Placebo signifikant um 18,9 % senkte. Es wurde die Einnahme von 2‑mal 0,5 mg Colchicin für 3 Tage, gefolgt von 0,5 mg/Tag für 27 Tage untersucht. Eine Pneumonie entwickelten 2,9 % der Patienten im Interventionsarm und 4,1 % der Patienten in der Placebogruppe [62].

Eine abschließende Beurteilung des Nutzens zu Colchicin zur COVID-19-Therapie ist aktuell anhand der vorhandenen Datenlage nicht möglich. Erste Daten legen einen potenziellen Nutzen des Medikamentes nahe, sollten jedoch mit Vorsicht betrachtet werden. Aufgrund der großen Heterogenität der Untersuchungsgruppen und des verschiedenen Studiendesigns sind größere randomisierte Studien essenziell für die Nutzenbeurteilung von Colchicin zur Therapie bei COVID-19.

Einen Überblick über die aktuelle Studienlage zu Colchicin (Stand 06.04.2021) gibt Tab. 3. Weitere 21 Studien zum Einsatz von Colchicin bei COVID-19 laufen aktuell noch und untersuchen den Nutzen des Medikamentes (Stand 06.04.2021) [63].

**Table 3:** 

Autoren	Design	Patientenzahl	Dosierung	Dauer	Outcome (Interventionsgruppe vs. Kontrollgruppe)	Bemerkung
Deftereos et al. (GRECCO-19)	Prospektiv, randomisiert, Open-Label kontrolliert Interventionsgruppe: Colchicin + Standardbehandlung Kontrollgruppe: Standardbehandlung	105 Interventionsgruppe: 50 Kontrollgruppe: 55	Sättigungsdosis 1,5 mg Colchicin p.o. (gefolgt von 0,5 mg 1 h später, bei Fehlen gastrointestinaler Nebenwirkungen); anschließend 0,5 mg Colchicin 2‑mal/Tag für maximal 3 Wochen bei gleichzeitiger Therapie mit Azithromycin: Sättigungsdosis 1 mg	03.04.–27.04.2020	*Klinische Effektivität:* Zeit bis zur klinischen Verschlechterung um 2 Punkte auf einer 7‑Grad-Skala^a^: 1,8 % vs. 14 % (*p* = 0,02) Mean ereignisfreies Überleben: 20,7 vs. 18,6 Tage (*p* = 0,03) *Biochemische Endpunkte:* hs-Troponin: 0,008 vs. 0,0112 (*p* = 0,34) Median CRP: 3,1 mg/dl vs. 4,5 mg/dl (*p* = 0,73) *Unerwünschte Ereignisse (UE):* Diarrhö: 45,5 % vs. 18 % (*p* = 0,003)	Median der maximalen D‑Dimer-Konzentration war statistisch signifikant niedriger in der Interventionsgruppe 0,76 [0,41 bis 1,59] μg/ml vs. 0,92 [0,68 bis 2,77] μg/m. (*p* = 0,04) Keine statistisch signifikanten Unterschiede in der Häufigkeit von weiteren UEs (Übelkeit, Erbrechen, abdominelle Schmerzen, Muskelspasmen, Kopfschmerzen)
Scarsi et al.	Kohortenstudie Interventionsgruppe: Colchicin + Standardbehandlung Kontrollgruppe: Standardbehandlung	262 Interventionsgruppe: 122 Kontrollgruppe: 140	Colchicin 1 mg/Tag Bei Diarrhö 0,5 mg/Tag	03/2020–04/2020	*Klinische Effektivität:* Niedrigeres Todesrisiko ist mit einer Behandlung mit Colchicin assoziiert (HR = 0,15 [95 %-CI 0,062 bis 0,368], *p* < 0,0001) Überlebensrate am 21. Tag: 84,2 % vs. 63,6 % Tod durch Komplikationen assoziiert mit COVID-19: 16,3 % vs. 37,1 % (*p* < 0,001)	Dosisreduktion bei 7,4 % der Patienten in der Interventionsgruppe wegen Diarrhö Keine Angaben zu weiteren UEs
Brunetti et al.	Retrospektive Beobachtungsstudie Interventionsgruppe: Colchicin + Standardbehandlung Kontrollgruppe: Standardbehandlung	66 Interventionsgruppe: 33 Kontrollgruppe: 33	Sättigungsdosis 1,2 mg, Erhaltungsdosis 2‑mal 0,6 mg/Tag	03/2020–05/2020	*Klinische Effektivität:* Tod im Krankenhaus am 28. Tag, unabhängig von der Ursache: 9,1 % vs. 33,3 % (*p* = 0,023) OSCI^b^ < 4 am Tag 14: 54,5 % vs. 54,5 % (*p* = 1) OSCI < 4 am Tag 21; 78,8 % vs. 54,5 (*p* = 0,04) Entlassene Patienten am Tag 28: 90,9 % vs. 66,7 (*p* = 0,02) *Biochemische Endpunkte:* CRP Reduktion von Baseline: 14,8 ± 9,1 mg/dl vs. 7,8 ± 6,0 mg/dl (*p* = 0,021)	CRP im Verlauf nur bei 9 Patienten untersucht, keine Angaben zu welchem Zeitpunkt Sättigungsdosis erhalten von 73 % der Patienten Keine Angaben zu UEs
Lopes et al.	Randomisiert, doppelt verblindet, placebokontrolliert Interventionsgruppe: Colchicin + Standardbehandlung Kontrollgruppe: Placebo + Standardbehandlung	75 Interventionsgruppe: 38 Kontrollgruppe: 37	Colchicin 0,5 mg 3‑mal/Tag über 5 Tage, gefolgt von 0,5 mg 2‑mal/Tag über 5 Tage Bei Gewicht > 80 kg, erste Dosis 1,0 mg Bei GFR < 30 ml/min/1,73 m^2^ – Dosisreduktion auf 0,25 mg 3‑mal/Tag über 5 Tage, dann 0,25 mg 2‑mal/Tag über 5 Tage	04/2020–08/2020	*Klinische Effektivität:* Dauer der Notwendigkeit einer Sauerstofftherapie: 4 Tage vs. 6,5 Tage (*p* < 0,001) Dauer der Hospitalisierung (Median): 7 Tage vs. 9 Tage (*p* = 0,003) Aufnahme auf Intensivstation: 5 % vs. 10,5 % Liegezeit auf Intensivstation: 12 vs. 11 Tage Bedarf an Sauerstofftherapie am 7. Tag: 9 % vs. 42 % (*p* = 0,001) *Biochemische Endpunkte:* CRP zeigte einen deutlicheren Abfall in der Interventionsgruppe (*p* = 0,001)	Keine statistische Analyse der Endpunkte „Aufnahme und Liegezeit auf Intensivstation“ wegen Seltenheit der Ereignisse Keine statistisch signifikanten Unterschiede in der Häufigkeit von UEs (Übelkeit, Diarrhö, Pneumonie, Serumspiegeln von Transaminasen)
Manenti et al.	Retrospektive Beobachtungsstudie Interventionsgruppe: Colchicin + Standardbehandlung Kontrollgruppe: Standardbehandlung	141 Interventionsgruppe: 70 Kontrollgruppe: 71	1 mg/Tag bis zur klinischen Verbesserung für maximal 21 Tage Bei Diarrhö und eGFR < 30 ml/min: 0,5 mg/Tag Dialysepatienten: 0,5 mg alle 2 Tage Patienten mit Leberinsuffizienz (bis Child-Pugh Score B): 0,5 mg alle 2 Tage	02/2020–04/2020	*Klinische Effektivität:* Kumulative Mortalität am 21. Tag: 7,5 % vs. 28,5 % (*p* = 0,006) Klinische Verbesserung am 21. Tag: 40 % vs. 26,6 % (*p* = 0,048) *Biochemische Endpunkte:* log2 CRP zeigte einen deutlicheren Abfall in der Interventionsgruppe (*p* = 0,009) Die Lymphozytenanzahl zeigte einen schnelleren Anstieg in der Interventionsgruppe (*p* = 0,018)	Diarrhö bei 2 Patienten, Hautekzem bei 2 Patienten in der Interventionsgruppe
Tardif et al. (COLCORONA)	Randomisiert, doppelt verblindet, placebokontrolliert Interventionsgruppe: Colchicin Kontrollgruppe: Placebo	4488 Interventionsgruppe: 2235 Kontrollgruppe: 2189	Colchicin 0,5 mg p.o. 2‑mal/Tag für die ersten 3 Tage, dann 1‑mal/Tag für die nächsten 27 Tage	03/2020–12/2020	*Klinische Effektivität:* Tod oder Hospitalisierung wegen COVID-19 30 Tage nach Randomisierung: 4,7 % vs. 5,8 % (*p* = 0,08) Hospitalisierung mit Notwendigkeit einer mechanischen Ventilation: 0,5 % vs. 1 % *Sicherheit und unerwünschte Ereignisse (UE):* Rate schwerer UEs 4,9 % vs. 6,3 % (*p* = 0,05) Pneumonie: 2,9 % vs. 4,1 % (*p* = 0,02) Lungenarterienembolie: 0,5 % vs. 0,1 % (*p* = 0,01) Diarrhö: 13,7 % vs. 7,3 % (*p* < 0,0001)	Nur Preprint des Manuskripts vom 26.01.2021 vorhanden (Stand 06.04.2021) Diagnosestellung mittels PCR (4159 Patienten) oder klinischen Kriterien (329)

^a^7‑Grad-Skala der klinischen Verschlechterung: (1) nicht hospitalisiert, normale Aktivität, (2) nicht hospitalisiert, Wiederaufnahme normaler Aktivitäten unmöglich, (3) hospitalisiert, ohne Sauerstofftherapie, (4) hospitalisiert unter Sauerstofftherapie, (5) hospitalisiert mit High-flow-Sauerstofftherapie, nichtinvasive Beatmung oder beide, (6) hospitalisiert, extrakorporale Membranoxygenierung, (7) Tod

^b^OSCI-Skala: (0) keine klinischen oder virologischen Zeichen einer Infektion, (1) keine Einschränkung der Aktivität, (2) eingeschränkte Aktivität, (3) hospitalisiert, ohne Sauerstofftherapie, (4) Sauerstofftherapie (Maske oder Nasenbrille), (5) nichtinvasive Beatmung der High-flow-Sauerstoff. (6) Intubation und mechanische Beatmung. (7) Beatmung inklusive zusätzliche Organunterstützung (Vasopressoren, Nierenersatztherapie, ECMO), (8) Tod

## Fazit für die Praxis

- Aufgrund seiner antiinflammatorischen Wirkung kommt Colchicin als Basismedikament zur Therapie verschiedener autoinflammatorischer Erkrankungen in der Rheumatologie zum Einsatz.
- Die häufigste unerwünschte Nebenwirkung der Colchicin-Therapie sind (dosisabhängige) Diarrhöen.
- Auch bei Kinderwunsch oder Schwangerschaft kann Colchicin weiter eingenommen werden. Schwangeren FMF-Patientinnen wird die Weitereinnahme sogar empfohlen.
- Eine vorsichtige Dosisanpassung des Colchicins ist bei Niereninsuffizienz zu beachten. Wechselwirkungen mit unerwünschter Toxizität können bei Komedikation mit Cytochrom P3A4- oder P‑Glykoprotein-Inhibitoren auftreten.
- Positive antiinflammatorische Effekte mit einer Verbesserung des klinischen Outcomes konnten auch in der Behandlung der koronaren Herzerkrankung in großen randomisierten Studien gezeigt werden. Colchicin könnte somit auch über die Rheumatologie hinaus in anderen Fachgebieten zum Einsatz kommen.
- Aktuell laufen u. a. auch mehrere Studien zum Einsatz von Colchicin bei COVID-19. Hier bleibt der Nutzen jedoch noch abzuwarten.
